# Endophytic Fungal Terpenoids: Natural Role and Bioactivities

**DOI:** 10.3390/microorganisms10020339

**Published:** 2022-02-01

**Authors:** Juan M. Galindo-Solís, Francisco J. Fernández

**Affiliations:** 1 Posgrado en Biotecnología, Universidad Autonoma Metropolitana, Unidad Iztapalapa, Mexico City CP 09340, Mexico; j.moises.galindo@gmail.com; 2 Departamento de Biotecnología, Universidad Autónoma Metropolitana, Unidad Iztapalapa, San Rafael Atlixco No. 186, Col. Vicentina, Mexico City CP 09340, Mexico

**Keywords:** bioactivity, chemotherapeutic, endophytic fungi, endophytism, terpenoids

## Abstract

Endophytic fungi are a highly diverse group of fungi that intermittently colonize all plants without causing symptoms of the disease. They sense and respond to physiological and environmental changes of their host plant and microbiome. The inter-organism interactions are largely driven by chemical networks mediated by specialized metabolites. The balance of these complex interactions leads to healthy and strong host plants. Endophytic strains have particular machinery to produce a plethora of secondary metabolites with a variety of bioactivities and unknown functions in an ecological niche. Terpenoids play a key role in endophytism and represent an important source of bioactive molecules for human health and agriculture. In this review, we describe the role of endophytic fungi in plant health, fungal terpenoids in multiple interactions, and bioactive fungal terpenoids recently reported from endophytes, mainly from plants used in traditional medicine, as well as from algae and mangroves. Additionally, we highlight endophytic fungi as producers of important chemotherapeutic terpenoids, initially discovered in plants. Despite advances in understanding endophytism, we still have much to learn in this field. The study of the role, the evolution of interactions of endophytic fungi and their terpenoids provide an opportunity for better applications in human health and agriculture.

## 1. Introduction

Endophytic fungi are a phylogenetically diverse group of fungi that asymptomatically and intermittently colonize plant tissue, driving saprophytic, commensalistic or mutualistic interaction with their host plant [1,2]. We can divide endophytic fungi into two groups: (1) Clavicipitaceous, represented by the genera *Epichloë* and *Neotyphodium*, and distinguished by a tight range of hosts and by mainly being transmitted vertically; (2) Nonclavicipitaceous, composed of a vast number of genera of Ascomycota, Mucormycota, and Basidiomycota [3]. The most commonly isolated genera include *Penicillium*, *Alternaria*, *Fusarium*, *Colletotrichum*, *Aspergillus*, and *Xylaria* [4]; nevertheless, the frequency of isolation depends on the plant species and genotypes, plant tissue samples, the geography of the plant, and the season of sampling [5,6,7,8]. Furthermore, endophytic strains are different from those that are non-endophytic given their different evolutionary and ecological context [5].

The endophytic fungal association is a complex chemical interkingdom interaction that involves the modulation of the defense mechanisms of the host plant and the regulation of fungal virulence factors, as well as antagonistic and cooperative interactions with other members of the microbiome [9]. A delicate balance between host plants with endophytic fungi and the rest of the microbiome leads to healthy plants [10]; moreover, endophytic fungi help to maintain plant health even under biotic and abiotic stress conditions [11,12]. As a consequence of these interactions, endophytic fungi produce a variety of secondary metabolites that can be exploited biotechnologically [11].

Terpenoids are the largest and diverse group of bioactive secondary metabolites. They perform important ecological functions, as defense and signaling molecules [13]. Endophytes biosynthesize a structurally and functionally diverse portfolio of terpenoids that act as long-distance communication among fungi and bacteria, insect attractors, antagonistic chemicals, plant growth promotors, or signaling molecules during symbiosis interaction [14]. Additionally, terpenoids are utilized as therapeutic drugs in human health. Indeed, remarkable drugs such paclitaxel, camptothecin, *Vinca alkaloids*, as well as fusidic acid, are natural terpenoid products [15]. Hundreds of new bioactive terpenoids have been isolated and characterized from endophytic fungi from sources such as plants used in traditional medicine, crops, algae, and mangroves [16].

Despite hundreds of publications on endophytic fungi that unquestionably place them as valuable sources of novel and known bioactive terpenoids, as well as recent advances in technology, fundamental questions remain unanswered in aspects such as plant–endophyte coevolution, horizontal gene transfer, metabolic pathways, and mechanisms underlying global interaction among endospheric and rhizospheric organisms. Therefore, integral studies, considering organisms at all levels and their interactions along with environmental aspects, have become indispensable.

## 2. Fungi Living Inside Plants

Initially, the term endophyte was used by De Bary, in 1866, to refer to all organisms that live within plant tissues [17]. During the study of endophytic tree leaf fungi, the term was defined to refer to fungi that live inside plants at some point in their life and that can colonize the internal tissues of plants without causing apparent damage, including latent pathogens that can live asymptomatically in their host for some time [18]. As the use of the term became popular and knowledge about these organisms increased, confusion and ambiguities arose in this regard [19]. In 1995, Wilson cleared up some confusion and misuse of the term. In this way, he highlighted that endophytic fungi colonize plant tissues without producing disease symptoms, defining them in terms of the nature of the plant–endophyte association [20]. Therefore, endophytic fungi include the entire spectrum of symbiotic interactions between plants and fungi: parasitism, commensalism, and mutualism [21]. Currently, we can highlight three characteristics of endophytes in terms of colonization of the plant: (1) they colonize the plant discreetly and for a long time; (2) colonization ceases temporarily; and (3) then, colonization resumes because of physical changes or modulators of the host plant. Whether the endophyte is subsequently considered a latent pathogen, a saprophyte, or a mutualist, episodic growth is a defining feature of endophytism [2]. To these characteristics, we can add that endophytic fungi have been found in all the main lineages of terrestrial plants, forming small ecosystems within them, and endophytic fungi strains are genetically different from pathogenic strains [1].

## 3. Endophytic Fungal Communities Are Diverse and Context-Dependent

As stated in the Introduction, the diversity of endophytic fungi includes two groups of fungi, the first of which is formed of members of the family *Clavicipitaceae*, a specific class of fungi that colonizes grasses as obligate biotrophic symbionts and can enhance the resistance of grasses to multiple stresses. *Epichloë* and *Neotyphodium* are remarkable genera of these clavicipitacoeus endophytic fungi. The second group, named nonclavicipitaceous, clusters phylogenetically diverse fungi from Ascomycota, Mucormycota, and Basidiomycota. Whereas the second group has a broad spectrum of hosts and is transmitted predominantly in a horizontal way, members of the first group have a reduced spectrum of hosts and are transmitted vertically [3,22]. In this review, we focus on the more frequent nonclavicipitaceous endophytic fungi.

Endophytic fungi are highly diverse phylogenetically and have been found in all land plants studied today. *Penicillium*, *Alternaria*, *Fusarium*, *Colletotrichum*, *Aspergillus*, and *Xylaria* are the most frequently isolated genera [4]; however, other reports have detected *Alternaria*, *Colletotrichum*, *Fusarium*, *Gibberella*, *Glomerella*, *Guignardia*, *Leptosphaerulina*, *Nigrospora*, *Phoma*, *Phomopsis*, and *Xylaria* [23]. It should be noted that the frequency of isolation of some genera depends on plant features, such as genotype, sampled tissue, geography, age, or season of sampling [24].

The diversity, specificity, and specialization of endophytic fungal communities are influenced both by geographic factors and by the genotype and ecological role of the host plant [5]. In a study with three native Hawaiian plants that co-occur along the elevation gradient (*Leptecophylla tameiameiae*, *Marchantia polymorpha*, and *Vaccinium reticulatum*), different patterns of endophytic fungi diversity, host specificity and specialization of interaction were observed at different elevations: less specialization and more diversity at the extremes of elevation. That is, at the extremes the associations were less specific, observing the greatest specialization at the mid-elevation [25]. Similarly, in the medicinal plant *Glycyrrhiza glabra* from different locations in the North-Western Himalayas, geographic location plays an important role in the recruitment of endophytic fungal communities, with the highest species richness being observed in the subtropical region [26]. Additionally, in this same genus of plant, the availability of potassium, nitrogen, and the accumulation of water and liquiritin in the roots influence the configuration of the structure of the endophytic fungal communities. In pines, each species has its own structure of endophytic fungi; in other words, the richness and structure of endophytic fungi vary depending on the pine space and the height of the trunk. The most commonly isolated genera were *Trichoderma*, *Penicillium*, *Aspergillus*, *Mucor*, *Alternaria*, *Sphaeropsis*, *Fusarium*, and *Chaetomium*. *Trichoderma* was common to all endophyte communities [27].

Although we can find certain species inhabiting several tissues of a host plant simultaneously, it is believed that these strains are genetically distinct, so the true diversity and specificity of endophytic fungal communities among different tissues can be observed at the genotype level.

Unlike roots, bark or phloem leaves are biochemically more dynamic as they are exposed to environmental damage and play a critical role in photosynthesis; as a consequence, foliar endophytes have important differences from root, bark, or phloem endophytes, since the colonization of leaves occurs in a substantially different context in comparison to roots, for example [5]. The analysis of 1400 foliar endophytic fungi from boreal to tropical forests revealed high species richness, observing few species and many classes of endophytes in boreal forests, as well as few classes but many species in tropical forests [28]. Evidence shows that the diversity and richness of species of foliar endophytes from trees are influenced by a variety of factors, for example, the age of host tree, with older trees having more diversity and richness of species. [29,30]. A recent study of the hyperdiversity of foliar endophytes from tropical forests showed that fungal richness decreased linearly with temperature seasonality, and as a quadratic function for precipitation seasonality, supporting the vital role of climate in shaping hyperdiversity of foliar endophytic fungi [31].

## 4. Endophytic Fungal Association: An Interkingdom Crosstalk

**Figure 1:**
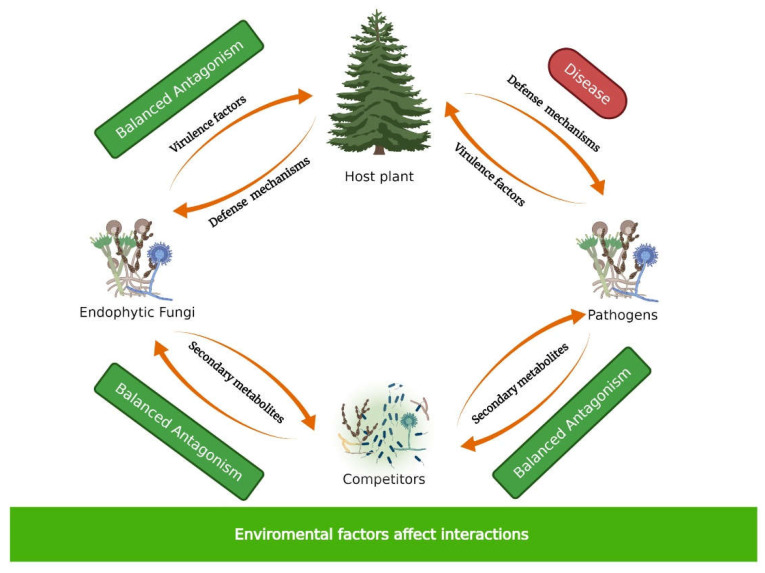
Multiple balanced antagonisms in endophytic fungi interactions. Created with BioRender.com, accessed on 3 September 2021.

Associations between plant and endophytic fungi involve a complex chemical interkingdom interaction including averting/suppressing the defense mechanisms of the host plant, the regulation of fungal virulence factors, and the mediation of coexistence with other fungi and bacteria inside the plant [32]. Thus, the host fungus downregulates some mechanisms and produces antagonistic secondary metabolites or chemical mediators to deal with competitors from the rest of the microbiome. This leads to multiple equilibria of balanced antagonisms and, therefore, contributes to the health of the host plant [33]. The plant–pathogen and plant–endophytic fungi interaction is the same during the first stages of contact; that is, both the pathogenic fungus and the endophyte produce virulence factors that will facilitate the colonization of the plant, while the plant responds with the defense mechanisms available. Unlike pathogenic fungi, which will continue to produce virulence factors until they colonize the plant and cause disease, endophytic fungi will modulate the production of virulence factors and will colonize the plant without disease symptoms (Figure 1). Additionally, the establishment of this association implies a process of recruitment of microbes by the host plant and inter- and intraspecies interactions to modulate the plant’s defense mechanisms [9]. The disequilibrium of the host plant–endophyte fungi relationship could compromise the health of the plant and become a pathogenic interaction [34,35]. The fate of the interaction is multifactorial, that is, whether the growth of the fungus is asymptomatic or will lead to disease depends on the adaptations of the fungus to a specific host or organ, on the endophyte and host states, on the innate but also variable endophyte virulence, the host defense response, and the environmental conditions [10,33,36]. Some observations have been made on the genomic level related to differences in the endophytic lifestyle evolution, for instance, comparative genomics of two Dark Septate Endophytes (DSE) (independently evolved in the same habitat) and 32 ascomycetes of different lifestyles showed that: first, DSE have larger genome sizes, in comparison with other ascomycetes, caused by expansion of the protein-coding gene inventory and expanded number of CAZymes, including plants cell wall degrading enzymes [37], in contrast to ectomycorrhizal fungi where the decay mechanisms are lost [38]; second, despite some similarities between the two DSE, low levels of convergence were observed in their gene family evolution, leading to marked functional heterogeneity within the endophytic lifestyle [37].

## 5. Endophytism and Pathogenicity

Many strains of endophytic fungi belong to genera and species of typically pathogenic or saprophytic plant fungi. In a variety of plants, their endophytes are capable of causing disease in the host plant when environmental conditions change. Such is the case of endophytic *Arabidopsis thaliana* fungi, which behave as pathogens when tested under in vitro conditions [6]. Similarly, in *Marchantia polymorpha*, although most of its endophytes promote growth, some are aggressively pathogenic. Even isolates of the same genus have very varied effects on the plant (positive, negative, or neutral) [39]. Single-species fungal populations may contain strains with pathogenic, mutualistic, or neutral behavior in their host [7]. For example, a study of 181 strains of the plant pathogenic fungus *Fusarium verticillioides*, isolated from corn plants at different locations and growth stages, showed that the growth cycle of the plant modulates the phytopathogenic behavior of the fungus, even behaving as a mutualistic endophyte. Regarding the characteristics of the isolated fungal populations, it was found that the phenological stages of corn act as a selection pressure for the fungi in favor of the plant [40]. This variability could be influenced by the genotypes of the host and the endophyte [41], the environmental conditions, the interactions among several endophytic fungi within the host plant [42], or by the presence of endobacteria or mycoviruses that can modulate the behavior of the fungus [43,44].

## 6. Endobacteria and Mycoviruses Can Modulate Endophytism

Endobacteria inhabiting endophytic and phytopathogenic fungi are associated with a positive effect on the performance of host fungi; in other words, endohyphal bacteria help to establish a pathogenic or symbiotic relationship with the host plant. [43]. For example, the symbiosis between the bacterium *Burkholderia rhizoxinica* and the phytopathogenic fungus *Rhizopus microsporus* leads to the production of rhizoxin, a potent antimitotic toxin that causes rice seedling blight [45], or rhizonin, a hepatotoxic cyclopeptide; both compounds help the fungus to set up infection [46]. A study revealed that *B. rhizoxinica* possesses a type III secretion system that is essential to maintain the bacterium–fungus symbiosis; moreover, the fungus strictly needs the bacterium for sporulation [47]. Further, recent research demonstrated that in addition to *B. rhizoxinica*-*R. microsporus* symbiosis, two narnaviruses are required for sexual reproductive success; in fact, narnaviruses decrease asexual reproduction of fungi [48]. In the case of endophytic fungi, a variety of endohyphal bacteria have been found in endophytic foliar fungi, mostly belonging to Proteobacteria [49]. The association of *Luteibacter* sp. with the endophytic fungus *Pestalotiopsis* aff. *neglecta* enhances the production of indole-3-acetic acid (IAA). During the culture of the pure bacterium, the presence of IAA was not observed [50].

The presence of mycoviruses is common in endophytic fungi and plant pathogenic fungi. These viruses are capable of regulating the virulence of phytopathogenic fungi (Table 1) [51]. This characteristic has been proposed as a promising strategy for the biological control of diseases in some crops. However, the use of these mycoviruses has been limited due to the little understanding that we still have about aspects such as transmission mechanisms, ecological impact, the effectiveness of their use in the field, and their persistence in the populations of fungi [44]. Recent studies have shown that mycoviruses, in addition to transforming pathogenic strains into hypovirulent ones, convert them into mutualistic endophytic fungi. As evidence, recently was demonstrated that the mycovirus SsHADV-1 from *Sclerotinia sclerotiorum*, a typically necrotrophic pathogen, converts the fungus into a beneficial endophyte [52]. Field experiments showed that the infected fungus promoted the growth of the host plant, improved its resistance to diseases and its yield. Similar behavior was observed with the pathogenic fungus *Pestalotiopsis theae* from *Camellia sinensis*: when it is infected with the mycovirus PtCV1, its pathogenicity is eliminated and the resistance of the host plant to the pathogenic strain of the fungus increases [53].

## 7. Secondary Metabolism and Endophytism

Unlike primary metabolites (proteins, carbohydrates, fats, and nucleic acids) that are essentially the same in all organisms, secondary metabolites are found in specific groups of organisms or species. Further, sometimes these molecules are produced under specific conditions; on the other hand, their specific function in an organism or ecological niche is often unknown [64]. However, some metabolites have well-known functions as competitive chemicals that act against other organisms, as metal transporting agents, growth stimulants, hormones or differentiation effectors [65]. Particularly, endophytic fungi are a reservoir of a variety of secondary metabolites with unknown natural functions but interesting bioactivities [66]. Concerning trophic interactions, secondary metabolites may have roles in establishing beneficial endophytic interaction with the host plant. For instance, genomic comparison of the beneficial root endophyte, *Colletotrichum tofieldiae*, and its pathogenic relative, *Colletotrichum incanum*, revealed significant enrichment genes encoding secondary metabolites, and biosynthesis-related proteins in *C. tofieldiae* [67]. Likewise, transcriptomic and quantitative real-time PCR of the endophyte *Phomopsis liquidambari* B3, during colonization and growth promotion of rice and *A. thaliana*, showed that secondary metabolite genes are u-regulated in the endophytic state [68]. More detailed investigation indicated that genes farnesyl-diphosphate farnesyl transferase and squalene monooxygenase were significantly upregulated. It should be noted that these genes are involved in sesquiterpenoid and triterpenoid biosynthesis [69], suggesting a key role in the secondary metabolism of endophytic fungi during early interactions with host plants.

## 8. Terpenoids in Fungal Interactions

Terpenoids represent structurally diverse molecules and as the largest group of natural products derived from C_5_ isoprene units, more than 80,000 terpenoids have been characterized from plant and microbial sources [70]. Biochemically, isoprene units may be derived through the mevalonic acid pathway (MVA) and methylerythritol phosphate pathway (MEP). According to their (C_5_)_n_ skeleton, they are classified as hemiterpenoids (C_5_), monoterpenoids (C_10_), sesquiterpenoids (C_15_), diterpenoids (C_20_), sesterterpenoids (C_25_), triterpenoids (C_30_), and tetraterpenoids (C_40_) [13,71]. Meroterpenoids are metabolites partially derived from terpenoids, for example, furocoumarins, retinoids, and ergot alkaloids [72].

The natural roles of terpenoids include antagonistic and beneficial interactions among organisms [71]. Particularly, fungal volatile terpenoids may play a key role in mediating endophytic fungi–host plant and endophytic fungi–microbiome interactions [14,73]. In general, volatile terpenoids are remarkable molecules interacting among different organisms. For example, fungal volatile sesquiterpenoids participate in the interaction with bacteria, other fungi, plants, and insects [14]. Patterns of fungal sesquiterpenoids and other volatile organic compounds (VOCs) emissions have been used to predict ecological function in several fungi (ectomycorrhizal, pathogens, and saprophytes) [74], supporting the role of terpenoids in fungal lifestyle [73].

**Figure 2:**
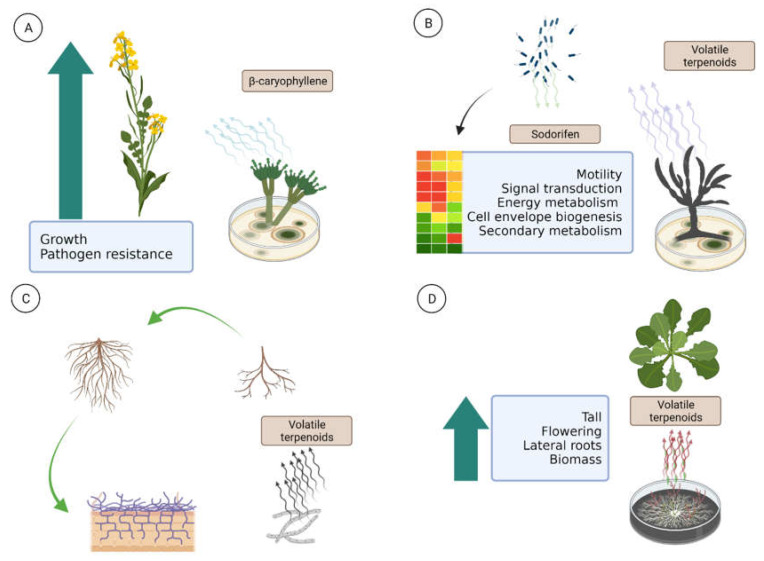
Natural role of fungal terpenoids. (**A**) Effects of fungal β-caryophyllene on *Brassica campestris*; (**B**) bacteria sensing and responding to fungal volatile terpenoids; (**C**) volatile terpenoids stimulating lateral root production; (**D**) effect of fungal terpenoids blend in *Arabidopsis*. Created with BioRender.com.

Volatile sesquiterpenoid β-caryophyllene is emitted by *A. thaliana* flowers and acts as a defense against pathogens [75]. Similarly, *Allium sativum* produces volatile terpenes with antifungal properties in response to infection with *Sclerotium cepivorum.* Additionally, *Talaromyces wortmannii* promotes growth and pathogen resistance of *Brassica campestris* L. var. *perviridis* by β-caryophyllene production (Figure 2A) [76]. In contrast to these results, neutral effects of β-caryophyllene in a mix of VOCs emitted by *Fusarium oxysporum* strains were observed in *A. thaliana* plants [77]. However, β-caryophyllene can induce global changes in the plant microbiome. For instance, *Bacillus amilolicuefaciens* induces the production of β-caryophyllene: as a consequence, host plants produce a large amount of salicylic acid. Interestingly, β-caryophyllene together with other VOCs modified the composition of rhizospheric microbes of surrounding plants [78]. Similarly, another work demonstrated that infected *Carex arenaria* plants produced VOCs in roots that stimulated the attraction of surrounding soil bacteria [79].

Other volatile terpenoids also modify the global metabolism of surrounding organisms. Metabolomic analysis of volatiles from *Fusarium culmorum* showed that several volatile terpenoids can induce or reduce the motility of *Collimonas pratensis* Ter291 and *Serratia plymuthica* PRI-2C [80]. Transcriptomic analysis revealed changes in gene and protein expression related to motility, signal transduction, energy metabolism, cell envelope biogenesis, and secondary metabolite production (Figure 2B). More detailed experiments exhibited bacterial production of sodorifen, an unusual volatile terpenoid [81]. Sodorifen is assumed to constitute the long-distance communication molecules among some bacteria [82]. These findings reveal that bacteria sense and respond to fungal volatile terpenoids and suggest the importance of volatiles as signaling molecules in fungal–bacterial interactions over long distances [80,81]. A similar role of monoterpenoids and sesquiterpenoids has been observed in plant–rhizosphere [83], bacteria–bacteria [84], and bacteria–protists interactions [85].

Some fungal terpenoids act as plant growth-promoting agents and as signaling molecules in the early stage of mutualistic interaction among plants and fungi. Typical ectomycorrhizal fungi, such as *Laccaria bicolor*, produce biologically active sesquiterpenoids while interacting with *Populus* sp. or *Arabidopsis* sp. plants, stimulating lateral root production (Figure 2C), an important pre-colonizing step occurring even before any physical contact between the fungus and the plant. More exhaustive experiments showed that sesquiterpenoid (–)-thujopsene can stimulate lateral root formation in absence of fungi, confirming the role of sesquiterpenoids in early mutualistic interactions [86]. Additionally, many volatile terpenoids function in specific mixes for promoting plants’ growth. For instance, *Trichoderma viride* produced 51 VOCs (among them seven sesquiterpenoids and one monoterpenoid) that positively influenced plant height, flowering, number of lateral roots, and biomass of *A. thaliana* (Figure 2D). Production of these compounds was independent of physical contact among plants and fungi [87]. A more detailed investigation revealed that VOCs of nine strains of *Trichoderma* sp. emitted more than 141 unique compounds, including several unknown sesquiterpenoids, diterpenoids, and tetraterpenoids. Blends of VOCs were strain-specific. Interestingly, biostimulatory strains tend to produce a larger number of complex terpenoids such as β-acoradiene, β-cubebene, β-cedrene, β-bisabolene, β-himachalene, and γ-himachalene [88].

Studies on endophytic fungal volatile terpenoids are still limited. Understanding the natural role and evolutionary aspects of these compounds will lead to applications of terpenoids to agriculture and ecosystem management [73,89].

## 9. Endophytic Fungal Terpenoids as Bioactive Molecules

The bioactivities of terpenoids include anticancer, anti-inflammatory, antibacterial, antiviral, and antimalarial effects, as well as hypoglycemic activities. Recent studies suggest their potential application as insect resistance, immunoregulation, antioxidation, antiaging, and neuroprotection agents [90]. Approximately one-third of all terpenoids included in the Dictionary of Natural Products (a compendium which provides properties and complete history of the relevant literature for over 328,000 natural compounds) have antineoplastic-related activities [91]. Despite the fact that many terpenoids show a variety of important activities, the molecular mechanisms of most terpenoids remain unclear; therefore, more studies on the mechanistic of activities are essential for a better understanding and application of these structurally diverse molecules [92]. In Table 2, Table 3, Table 4 and Table 5 below, we summarize the bioactive terpenoids isolated from endophytic fungi of medicinal plants, mangroves, algae, and some trees, mosses, and crops reported from 2017 to the first third of 2021 (Table 2, Table 3, Table 4 and Table 5).

Although a three-year period may be too short to obtain a representative analysis, Basidiomycota are the most abundant endophytic fungi producing bioactive terpenoids in this review (97.4%). They were grouped in 13 orders: *Eurotiales* (40%), *Hypocreales* (16%), *Diaporthales* (12%), *Pleosporales* (12%), *Botryosphaeriales* (10.7%), *Xylariales* (4%), *Glomerellales* (2.7%), *Chaetothyriales* (1.3%), *Helotiales* (1.3%), *Microascales* (1.3%), *Pezizales* (1.3%), *Togniniales* (1.3%), and *Venturiales* (1.3%). The most frequently isolated fungi belong to *Eurotriales*, being *Aspergillus* and *Penicillium* the predominant species. In addition, 50% of all species in this review are included in two families, *Trichocomaceae* and *Hypocreaceae.*

## 10. Endophytic Fungi Biosynthesize Important Therapeutic Drugs

In general, microbes produce a collection of secondary metabolites with therapeutic activities. Many are used as anticancer, immunosuppressive, hypocholesterolemic, antiparasitic, anti-inflammatory agents, or as enzyme inhibitors [161,162]. Indeed, endophytic fungi have become a treasure trove for bioactive compounds of medicinal and agricultural importance [163]. Several endophytic fungi produce important therapeutic drugs, initially discovered in traditional medicinal plants (Table 6); for example, paclitaxel, podophyllotoxin, *Vinca alkaloids*, camptothecin, and fusidic acid [163].

### 10.1. Paclitaxel

Paclitaxel is possibly the most famous natural product of endophytic fungi. This highly functionalized diterpenoid is a potent antimitotic compound originally isolated from the stem bark of the western yew, *Taxus brevifolia*. It was the first natural substance that demonstrated antimycotic, antileukemic, and tumor inhibitory activities [164]. In 1993, the first endophytic fungi producing paclitaxel was reported. *Taxomyces andreanae* was isolated from the inner bark of *T. brevifolia* and produced paclitaxel and related compounds when grown in a semisolid synthetic medium [165]. Frequently, paclitaxel-producing endophytic fungi have been isolated from different sources other than *Taxus* trees, even at a higher concentrations than those isolated from *Taxus* trees; such is the case of studies focused on endophytes from plants used in traditional medicine [166,167,168,169]. For example, *Cladosporium oxysporum*, isolated from the medicinal plant *Moringa oleifera* yields 550 μg/L in liquid fermentation [170], a high concentration considering that *Taxaomyces andrenae* yielded 0.05 μg/L at similar culture conditions. Likewise, *Phoma betae* from Ginkgo biloba leaves yielded 795 μg/L [168]. Now, hundreds of fungi isolated from yew and other plants have been shown to produce paclitaxel [171], including patents for the production of paclitaxel from endophytic fungi focused on optimization of the production process, methods for purification from the fermentation broth, and methods for screening paclitaxel-producing endophytic fungi [172].

### 10.2. Podophyllotoxin

Podophyllotoxin is an aryltetralin–lignan anticancer metabolite produced by several plants, mainly by *Sinopodophyllum hexandrum*; it is used in the East and Middle East as traditional medicine. Podophyllotoxin serves as a precursor to three key chemotherapeutic drugs: etoposide, teniposide, and etoposide phosphate [173]. Etoposide is widely used to treat various types of cancer and has recently been proposed as an adjunct treatment to immunosuppressants for critically ill COVID-19 patients [174,175]. Several endophytic fungi from different plant species have been reported to produce podophyllotoxin at different concentrations; likewise, two patents concerning methods for the identification of podophyllotoxin-producing fungi and production and recuperation processes of podophyllotoxin in liquid fermentation have been issued [172].

### 10.3. Vinca alkaloids

*Vinca alkaloids* (vincristine and vinblastine) and semisynthetic derivatives (vinorelbine, vindesine, and vinflunine) are remarkable antimitotic chemotherapeutics utilized in the treatment of hematological and lymphatic neoplasms. These indole terpenoids stop mitosis by inhibiting the formation of microtubules (at low concentration) or depolymerizing microtubules (at high concentrations) [176]. Some fungi produce vinblastine or vincristine under specific culture conditions, such as *Botryosphaeria laricina* CRS1, an endophyte of *Catharanthus roseus*, in which high yields of vinblastine and vincristine are dependent on elicitors present in extracts of the host plant [177]. In the case of *Alternaria alternata and Talaromyces radius*, the yield of vinblastine depends on media composition [178,179]. Despite the fact that *Catharanthus roseus* has many endophytic fungi, only some of them have been demonstrated to produce vinblastine or vincristine [180,181]. Other approaches include the use of endophytes to elicit the accumulation of *Vinca alkaloids* in the leaves of *C. roseus*. Inoculation of these plants with two of their endophytes (*Curvularia* sp. CATDLF5 and *Choanephora infundibulifera* CATDLF6) was found to enhance vindoline content by upregulating genes related to the terpenoid indole alkaloid biosynthesis in *C*. *roseus* [182]. Similar results were observed in cell suspension cultures of the same plant [183].

### 10.4. Camptothecin

Together with paclitaxel and *Vinca alkaloids*, camptothecin (including its analogs) belongs to the most important chemotherapeutic drugs. Camptothecin is an indole-terpene alkaloid extracted from the bark of *Camptotheca acuminata* [184], used as the precursor of two more potent camptothecin analogs: topotecan, and irinotecan. Different from the previously described substances, which are antimitotic drugs, camptothecins belong to the group of topoisomerase inhibitors, particularly camptothecins that act by inhibiting DNA topoisomerase I, an enzyme found in significantly high levels in many cancer surgical specimens [176,185]. Commercially, camptothecin is extracted from *C. acuminata* and *Nothapodytes nimmoniana* with yields up to 0.3% of dry weight [186]. Most endophytic camptothecin-producing fungi have been isolated from those host plants (Table 6). *Aspegillus niger*, *Alternaria alternata*, and *Fusarium solani* were isolated from *Piper betle*, *Miquelia dentata*, and *Apodytes dimidiata*, respectively [187,188,189]. The highest yield was obtained from *Trichoderma atroviride* LY357, isolated from *C. acuminata*, about 197.82 µg/L [190]. Despite the high yield among endophytic fungi, it is little exploited in industry [191].

### 10.5. Fusidic Acid

Fusidic acid is an antibiotic isolated in 1962 from the fermentation broth of a strain of *Fusidium coccineum* [192]. Chemically, it is a fusidane triterpenoid inhibitor of prokaryotic elongation factor (EF-G), hence it stops protein synthesis [193]. This antibiotic is particularly important in infections by staphylococci, including the methicillin-resistant *Staphylococcus aureus* (MRSA) [194]. Recently, the endophytic fungus *Acremonium pilosum* F47 has been reported to produce authentic fusidic acid, two known analogs (16-desacetylfusidic acid and 3β,20-dihydroxy-protosta-16,24-dien-29-oic acid), and a new derivative, acremonidiol A. [195]. A few more fungi, such as *Sarocladium oryzae*, an endophyte of *Oryza rufipogon* Griff. (Dongxiang wild rice) [196], and *Xylaria* sp., endophyte of *Anoectochilus setaceus* [197], have been shown to produce other fusidane-type antibiotics, including helvolic acid.

**Table 6:** Endophytic fungi that produce important therapeutic drugs.

Secondary Metabolite	Representative Endophytic Fungi	References
**Paclitaxel (anticancer chemotherapy drug)**	*Aspergillus candidus*, *Chaetomella raphigera*, *Cladosporium cladosporioides*, *Cladosporium oxysporum*, *Lasiodiplodia theobromae*, *Penicillium aurantiogriseum*, *Periconia* sp., *Pestalotiopsis microspora*, *Pestalotiopsis versicolor*, *Phoma betae*, *Phomopsis* sp., *Phomopsis* sp., *Phomopsis* sp., *Phyllosticta citricarpa*, *Phyllosticta melochiae*	[168,169,170,198,199,200,201,202,203,204,205,206,207]
**Camptothecin and analogs (anticancer chemotherapy drug)**	*Fusarium solani*, *Fusarium oxysporum*, *Entrophospora infrequens*, *Trichoderma atroviride*, *Neurospora* sp., *Alternaria alstroemeriae*, *Alternaria burnsii*, *Alternaria* sp., *Alternaria alternata*, *Xylaria* sp., *Aspergillus* sp., *Aspergillus niger*	[187,188,189,190,208,209,210,211,212,213,214,215]
**Vinblastine and vincristine (anticancer chemotherapy drug)**	*Alternaria alternata* sp, *Fusarium oxysporum*, *Talaromyces radicus*, *Curvularia verruculosa*, *Botryosphaeria laricina*	[177,178,179,216,217]
**Podophyllotoxin (anticancer chemotherapy)**	*Phialocephala fortinii (0.5 to 189 μg/L)*, *Alternaria tenuissima*, *Mucor fragilis*, *Trametes hirsuta*, *Alternaria* sp. *Fusarium solani*	[218,219,220,221,222,223]
**Fusidic acid (antibiotic)**	*Acremonium pilosum*	[195]

## 11. Challenges and Future Perspectives

Although many endophytic fungal strains reach relatively high yields, and there are at least 28 patents available regarding the production of pharmaceutically important secondary metabolites [172], complex challenges remain related to the poor understanding of the biochemical, molecular, and evolutionary principles driving the biosynthesis of bioactive molecules [224].

Many limitations for the use of secondary metabolites from endophytic fungi stem from poor understanding of endophytism. As a consequence, we do not clearly understand the ecological role of most secondary metabolites, and questions emerge regarding how biosynthesis of certain metabolites is regulated. Does this regulation depend only on the endophyte or the host plant? Or both? How do environmental factors, such as biotic and abiotic stress, influence the production of a specific secondary metabolite? All these questions are key during the design of industrial production processes [225,226]. More generally, our knowledge and understanding of the biology of many fungi is limited. Therefore, there are limited numbers of molecular and synthetic biology tools, high-throughput technologies, and high-quality annotated and curated fungal genomes [227]. As noted above, challenges in the use of bioactive terpenoids and other secondary metabolites are complex, thus, more multidisciplinary research in fungal biology and biotechnology is needed.

## Figures and Tables

**Table 1:** Effect of viruses in fungi.

Host Plants	Endophytic/Pathogenic Fungi	Mycoviruses	Effect in Host Plants	References
***Camellia sinensis***	*Pestalotiopsis theae*	chrysovirus-1	Eliminates fungal virulence and confers disease resistance in plants	[53]
***Castanea dentata***	*Cryphonectria parasitica*	Cryphonectria hypovirus 1 (CHV1), Cryphonectria parasitica mitovirus 1 (CpMV1)	Hypovirulence	[54,55]
**More than 64 genera of plants**	*Sclerotinia sclerotiorum*	Sclerotinia sclerotiorum hypovirulence-associated DNA virus 1 (SsHADV-1), Hubei sclerotinia RNA virus 1 (HuSRV1)	Hypovirulence, growth promotion, disease resistance, and improved yield of host plants	[52,56,57]
***Cucumis sativus*** **, *Fragaria × ananassa*, *Vitis vinifera*, *Lycopersicon esculentum***	*Botrytis cinerea*	Botrytis cinerea partitivirus 2 (BcPV2), Botrytis cinerea fusarivirus 1 (BcFV1)	Hypovirulence	[58,59]
***Zea mays***	*Fusarium graminearum*	Double-stranded RNA (dsRNA)	Hypovirulence	[60]
***Dichanthelium lanuginosum***	*Curvularia protuberia*	Curvularia thermal tolerance virus (CThTV)	Confers heat tolerance to host plant	[61,62]
***Brassica napus***	*Leptosphaeria biglobosa*	Double-stranded RNA quadrivirus	Hypovirulence, enhances systemic resistance to *Leptosphaeria maculans*	[63]

**Table 2:** Bioactive terpenoids isolated from endophytic fungi of medicinal plants.

Host Plant	Endophyte	Bioactivity	Class of Terpenoid	References
***Phyllanthus glaucus***	*Phomopsis* sp. TJ507A	Inhibition of BACE1	Sesquiterpenoid	[93]
***Hypericum ascyron***	*Phomopsis prunorum*	Antibacterial		[94]
***Ligusticum wallichii***	*Aspergillus fumigatus*	Cytotoxic		[95]
***Artemisia argyi***	*Trichoderma virens* QA-8	Antimicrobial		[96]
***Tylophora ovata***	*Aspergillus flavus*	Cytotoxic and hepatic protection effects		[97]
***Panax notoginseng***	*Leptosphaeria* sp. XL026	Antibacterial		[98]
***Aconitum vilmorinianum***	*Phoma* sp.	Antiviral (H1N1)		[99]
***Morinda officinalis***	*Trichoderma koningiopsis* A729	Antibacterial and cytotoxic		[100]
***Edgeworthia chrysantha***	*Penicillium purpurogenum*	Inhibition of pancreatic lipase		[101]
***Panax notoginseng***	*Emericella* sp. XL 029	Antimicrobial		[102]
***Panax notoginseng***	*Preussia isomera*	Antibacterial		[103]
***Panax notoginseng***	*Leptosphaeria* sp. XL026	Antibacterial	Diterpenoid	[98]
***Huperzia serrata***	*Penicillium chrysogenumm* MT-12	Inhibition of activity on ATP release of thrombin-activated platelets		[104]
***Illigera rhodantha***	*Phomopsis* sp. S12	Anti-inflammatory		[105]
***Colquhounia coccinea var. mollis***	*Trichoderma atroviride*	Antibacterial activity and cytotoxic		[106]
***Zingiber officinale***	*Penicillium* sp. ZO-R1-1	Cytotoxic		[107]
***Panax notoginseng***	*Drechmeria sp*	Antimicrobial		[108]
***Gliricidia sepium***	*Nectria pseudotrichia* 120-1NP	Cytotoxic		[109]
***Morinda officinalis***	*Trichoderma koningiopsis* A729	Antibacterial and cytotoxic		[100]
***Clerodendrum canescens***	*Pestalotiopsis adusta*	Cytotoxic		[110]
***Salvia miltiorrhiza***	*Talaromyces pinophilus*	Antimicrobial activity		[111]
***Lycium barbarum***	*Strain L1-2*	Cytotoxic	Meroterpenoid	[112]
***Hypericum perforatum***	*Aspergillus* sp. TJ23	Potentiator of oxacillin		[113]
***Hypericum perforatum***	*Aspergillus* sp. TJ23	Antibacterial Potentiator of oxacillin		[114]
***I. eriocalyx var. laxiflora***	*Penicillium* sp. sh18	Cytotoxic. Inhibition of tubulin polymerization		[115,116]
***Acorus tatarinowii***	*Phyllosticta* sp.	Antimicrobial		[117]
***Tripterygium wilfordii***	*Aspergillus* sp.	Immunosuppressive		[118]
***Handroanthus impetiginosus***	*Talaromyces purpurogenus* H4 *and Phanerochaete* sp. H2	Trypanocidal		[119]
***Tripterygium wilfordii***	*Aspergillus terreus Thom*	Inhibition of BACE-1 and AchE		[120]
***Hypericum perforatum***	*Emericella* sp. TJ29	Cytotoxic		[121]
***Withania somnifera***	*Colletotrichum gigasporum*	Inhibition of pancreatic lipase	Triterpenoid	[122]
***Centella asiatica (L.) Urban***	*Colletotrichum gloeosporioides*	Cytotoxic and immunomodulatory		[123]
***Kadsura angustifolia***	*Trichoderma harzianum* SWUKD3.1610	Inhibition of HIV-1 Reverse Transcriptase and Cytotoxic		[124]
***Zingiber cgriffithii Baker***	*Hypomontagnella monticulosa* Zg15SU	Cytotoxic	Sesterterpenoid	[125]
***Panax notoginseng***	*Preussia isomera*	Antibacterial		[103]
***Paeonia lactiflora Pallas***	*Alternaria tenuissima*, *Aspergillus flavus*, *and Penicillium commune)*	Anti-inflammatory	Monoterpenoid	[126,127]
***Ferula ovina***	*Pithoascus persicus*, *Ochroconis ferulica*, *Alternaria petroselini*, *Lasiobolidium* sp. *Nov.*, *Clonostachys rosea*, *Laburnicola* sp. *Nov.*, *Phaeoacremonium* sp. *Cadophora interclivum*	Cytotoxic		[128,129]

**Table 3:** Bioactive terpenoids isolated from endophytic fungi of mangrove.

Host Plant	Endophyte	Bioactivity	Class of Terpenoid	References
***Acrostichum aureum***	*Rhinocladiella similis*	Cytotoxic	Sesquiterpenoid	[130]
***Kandelia obobata***	*Aspergillus flavus* QQSG-3	Inhibition of α-glucosidase		[131]
***Ceriops tagal***	*Penicillium* sp. J-54	Cytotoxic		[132]
***Kandelia candel***	*Diaporthe* sp. QYM12	Anti-inflammatory		[133]
***Xylocarpus moluccensis***	*Aspergillus* sp. xy02	Antibacterial		[134]
***Ceriops tagal***	*Cytospora* sp.	Antimicrobial		[135]
***Kandelia candel***	*Diaporthe* sp. QYM12	Anti-inflammatory	Diterpenoid	[133]
***Kandelia obovate***	*Talaromyces amestolkiae* YX1	Anti-inflammatory activity	Meroterpenoid	[136]
***Sonneratia apetala***	*Aspergillus* sp. 16-5c	Inhibition of AchE		[137]
***Kandelia obovata***	*Aspergillus terreus* H010	Anti-inflammatory		[138]
***Bruguiera sexangula***	*Phyllosticta capitalensis*	Antimicrobial		[139]
***Kandelia cande***	*Aspergillus* sp. ZJ-68	Anti-inflammatory and inhibition of PtpB from *Mycobacterium tuberculosis*	Sesterterpenoid	[140]

**Table 4:** Bioactive terpenoids isolated from endophytic fungi of alga.

Host Plant	Endophyte	Bioactivity	Class of Terpenoid	References
***Laminaria japonica***	*Trichoderma harzianum* X-5	Antiphytoplankton	Sesquiterpenoid	[141]
***Pterocladiella capillacea***	*Chondrostereum* sp. NTOU4196	Anti-inflammatory in microglial		[142]
***Sargassum* sp.**	*Trichoderma asperellum* cf44-2	Antibacterial and Antiphytoplankton		[143]
***Laminaria japonica***	*Trichoderma harzianum* X-5	Antiphytoplankton	Diterpenoid	[141]
***marine-derived***	*Aspergillus porosus*	Antibacterial		[144]
***Leathesia nana***	*Penicillium chrysogenum* XNM-12	Antimicrobial	Meroterpenoid	[145]
***Rhodomela confervoides***	*Aspergillus* sp. RR-YLW-1	Antimicroalgae		[146]
***Rhodomela confervoides***	*Aspergillus* sp. RR-YLW-1	Antimicroalgae	Sesterterpenoid	[146]
***Sargassum* sp.**	*Trichoderma asperellum* cf44-2	Antibacterial and Antiphytoplankton	Monoterpenoid	[143]

**Table 5:** Bioactive terpenoids isolated from endophytic fungi of diverse plants.

Host Plant	Endophyte	Bioactivity	Class of Terpenoid	References
***Elaeocarpus decipiens Hemsl* (tree)**	*Aspergillus versicolor*	Antimicrobial	Sesquiterpenoid	[147]
***Oxytropis glabra* (desert plant)**	*Alternaria oxytropis*	Retarded seedling growth of *Arabidopsis thaliana*	Sesquiterpenoid	[148]
***Rhodobryum umgiganteum* (moss)**	*Botrysphaeria laricina*	Induction of quinone reductase	Diterpenoid and meroterpenoid	[149,150,151]
***Toona sinensis* (tree)**	*Xylaria* sp. XC-16	Allelopathic	Diterpenoid	[152]
***Cephalotaxus fortune* (tree)**	*Phyllosticta capitalensis*	Phytotoxic	Meroterpenoid	[153]
***Dendrobium nobile* (ornamental)**	*Guignardia mangiferae* TJ414	Anti-inflammatory	Meroterpenoid	[154]
***Kageneckia angustifolia* (Ornamental)**	*Penicillium* sp. SWUKD4.1850	Cytotoxic	Triterpenoid	[155]
***Polytrichum commune* (moss)**	*Diplodia cupressi*	Cytotoxic	Triterpenoid	[156]
***Laptospermum brachyandrum* (tree)**	*Eutypella scoparia* SCBG-8	Antibacterial (MRSA)	Triterpenoid	[157]
***Triticum* (crop)**	*Bipolaris* sp. TJ403-B1	Antimicrobial and anti-inflammatory	Sesterterpenoid	[158,159]
***Cucumis sativus* (crop)**	*Paecilomyces formosus* LHL10	Inhibition of α-glucosidase and urease	Sesterterpenoid	[160]

## References

[1] Schouten A., Schouten A. (2019). Endophytic fungi: Definitions, diversity, distribution and their significance in plant life. Endophyte Biotechnology: Potential for Agriculture and Pharmacology.

[2] Stone J.K., Bacon C.W., White J.F., Mueller G.M., Bills G.F., Foster M.S. (2005). Endophytic fungi. Biodiversity of Fungi: Inventory and Monitoring Methods.

[3] Rodriguez R.J., White J.F., Arnold A.E., Redman R.S. (2009). Fungal endophytes: Diversity and functional roles. New Phytol..

[4] Rashmi M., Kushveer J., Sarma V. (2019). A worldwide list of endophytic fungi with notes on ecology and diversity. Mycosphere.

[5] Arnold A.E. (2007). Understanding the diversity of foliar endophytic fungi: Progress, challenges, and frontiers. Fungal Biol. Rev..

[6] Junker C., Draeger S., Schulz B. (2012). A fine line—Endophytes or pathogens in *Arabidopsis thaliana*. Fungal Ecol..

[7] Brader G., Compant S., Vescio K., Mitter B., Trognitz F., Ma L.-J., Sessitsch A. (2017). Ecology and genomic insights into plant-pathogenic and plant-nonpathogenic endophytes. Annu. Rev. Phytopathol..

[8] Fisher P.J., Petrini O. (1987). Location of fungal endophytes in tissues of *Suaeda fruticosa*: A preliminary study. Trans. Br. Mycol. Soc..

[9] Chagas F.O., de Cassia Pessotti R., Caraballo-Rodríguez A.M., Pupo M.T. (2018). Chemical signaling involved in plant–microbe interactions. Chem. Soc. Rev..

[10] Schulz B., Haas S., Junker C., Andrée N., Schobert M. (2015). Fungal endophytes are involved in multiple balanced antagonisms. Curr. Sci..

[11] Eid A.M., Salim S.S., Hassan S.E.-D., Ismail M.A., Fouda A., Kumar V., Prasad R., Kumar M., Choudhary D.K. (2019). Role of endophytes in plant health and abiotic stress management. Microbiome in Plant Health and Disease: Challenges and Opportunities.

[12] Terhonen E., Blumenstein K., Kovalchuk A., Asiegbu F.O. (2019). Forest tree microbiomes and associated fungal endophytes: Functional roles and impact on forest health. Forests.

[13] Dewick P.M. (2009). The mevalonate and methylerythritol phosphate pathways: Terpenoids and steroids. Medicinal Natural Products.

[14] Kramer R., Abraham W.-R. (2012). Volatile sesquiterpenes from fungi: What are they good for?. Phytochem. Rev..

[15] Uzma F., Mohan C.D., Hashem A., Konappa N.M., Rangappa S., Kamath P.V., Singh B.P., Mudili V., Gupta V.K., Siddaiah C.N. (2018). Endophytic fungi—Alternative sources of cytotoxic compounds: A review. Front. Pharmacol..

[16] Souza J.J.d., Vieira I.J.C., Rodrigues-Filho E., Braz-Filho R. (2011). Terpenoids from endophytic fungi. Molecules.

[17] Hide K.D., Soytong K. (2008). The fungal endophyte dilemma. Fungal Divers..

[18] Petrini O. (1991). Fungal endophytes of tree leaves. Microbial Ecology of Leaves.

[19] Wennström A. (1994). Endophyte: The misuse of an old term. Oikos.

[20] Wilson D. (1995). Endophyte: The evolution of a term, and clarification of its use and definition. Oikos.

[21] Stone J.K., Bacon C.W., White J.F. (2000). An overview of endophytic microbes: Endophytism defined. Microbial Endophytes.

[22] Kuldau G., Bacon C. (2008). Clavicipitaceous endophytes: Their ability to enhance resistance of grasses to multiple stresses. Biol. Control.

[23] Zheng Y.-K., Qiao X.-G., Miao C.-P., Liu K., Chen Y.-W., Xu L.-H., Zhao L.-X. (2016). Diversity, distribution and biotechnological potential of endophytic fungi. Ann. Microbiol..

[24] Rana K.L., Kour D., Sheikh I., Yadav N., Yadav A.N., Kumar V., Singh B.P., Dhaliwal H.S., Saxena A.K., Singh B.P. (2019). Biodiversity of endophytic fungi from diverse niches and their biotechnological applications. Advances in Endophytic Fungal Research: Present Status and Future Challenges.

[25] Cobian G.M., Egan C.P., Amend A.S. (2019). Plant–microbe specificity varies as a function of elevation. ISME J..

[26] Arora P., Wani Z.A., Ahmad T., Sultan P., Gupta S., Riyaz-Ul-Hassan S. (2019). Community structure, spatial distribution, diversity and functional characterization of culturable endophytic fungi associated with *Glycyrrhiza glabra* L.. Fungal Biol..

[27] Wang L., Ren L., Li C., Gao C., Liu X., Wang M., Luo Y. (2019). Effects of endophytic fungi diversity in different coniferous species on the colonization of *Sirex noctilio* (Hymenoptera: Siricidae). Sci. Rep..

[28] Arnold A.E., Lutzoni F. (2007). Diversity and host range of foliar fungal endophytes: Are tropical leaves biodiversity hotspots?. Ecology.

[29] Arnold A.E., Mejía L.C., Kyllo D., Rojas E.I., Maynard Z., Robbins N., Herre E.A. (2003). Fungal endophytes limit pathogen damage in a tropical tree. Proc. Natl. Acad. Sci. USA.

[30] Yu Z., Ding H., Shen K., Bu F., Newcombe G., Liu H. (2021). Foliar endophytes in trees varying greatly in age. Eur. J. Plant Pathol..

[31] Oita S., Ibáñez A., Lutzoni F., Miadlikowska J., Geml J., Lewis L.A., Hom E.F., Carbone I., U’Ren J.M., Arnold A.E. (2021). Climate and seasonality drive the richness and composition of tropical fungal endophytes at a landscape scale. Commun. Biol..

[32] Mattoo A.J., Nonzom S. (2021). Endophytic fungi: Understanding complex cross-talks. Symbiosis.

[33] Schulz B.J., Rabsch L., Junker C., Verma S.K., White J.J.F. (2019). Chemical warfare in the plant microbiome leads to a balance of antagonisms and a healthy plant. Seed Endophytes: Biology and Biotechnology.

[34] Steinert M., Hentschel U., Hacker J. (2000). Symbiosis and pathogenesis: Evolution of the microbe-host interaction. Naturwissenschaften.

[35] Kogel K.-H., Franken P., Hückelhoven R. (2006). Endophyte or parasite—What decides?. Curr. Opin. Plant Biol..

[36] Schulz B., Boyle C. (2005). The endophytic continuum. Mycol. Res..

[37] Knapp D.G., Németh J.B., Barry K., Hainaut M., Henrissat B., Johnson J., Kuo A., Lim J.H.P., Lipzen A., Nolan M. (2018). Comparative genomics provides insights into the lifestyle and reveals functional heterogeneity of dark septate endophytic fungi. Sci. Rep..

[38] Kohler A., Kuo A., Nagy L.G., Morin E., Barry K.W., Buscot F., Canbäck B., Choi C., Cichocki N., Clum A. (2015). Convergent losses of decay mechanisms and rapid turnover of symbiosis genes in mycorrhizal mutualists. Nat. Genet..

[39] Nelson J.M., Hauser D.A., Hinson R., Shaw A.J. (2018). A novel experimental system using the liverwort *Marchantia polymorpha* and its fungal endophytes reveals diverse and context-dependent effects. New Phytol..

[40] Venturini G., Assante G., Toffolatti S.L., Vercesi A. (2013). Pathogenicity variation in *Fusarium verticillioides* populations isolated from maize in Northern Italy. Mycoscience.

[41] Mandyam K.G., Roe J., Jumpponen A. (2013). *Arabidopsis thaliana* model system reveals a continuum of responses to root endophyte colonization. Fungal Biol..

[42] Aguilar-Trigueros C.A., Rillig M.C. (2016). Effect of different root endophytic fungi on plant community structure in experimental microcosms. Ecol. Evol..

[43] Bastías D.A., Johnson L.J., Card S.D. (2020). Symbiotic bacteria of plant-associated fungi: Friends or foes?. Curr. Opin. Plant Biol..

[44] Muñoz-Adalia E.J., Fernández M.M., Diez J.J. (2016). The use of mycoviruses in the control of forest diseases. Biocontrol Sci. Technol..

[45] Scherlach K., Partida-Martinez L.P., Dahse H.-M., Hertweck C. (2006). Antimitotic rhizoxin derivatives from a cultured bacterial endosymbiont of the rice pathogenic fungus *Rhizopus microsporus*. J. Am. Chem. Soc..

[46] Partida-Martinez L.P., Looß C.F.d., Ishida K., Ishida M., Roth M., Buder K., Hertweck C. (2007). Rhizonin, the first mycotoxin isolated from the Zygomycota, is not a fungal metabolite but is produced by bacterial endosymbionts. Appl. Environ. Microbiol..

[47] Lackner G., Moebius N., Hertweck C. (2011). Endofungal bacterium controls its host by an *hrp* type III secretion system. ISME J..

[48] Espino-Vázquez A.N., Bermúdez-Barrientos J.R., Cabrera-Rangel J.F., Córdova-López G., Cardoso-Martínez F., Martínez-Vázquez A., Camarena-Pozos D.A., Mondo S.J., Pawlowska T.E., Abreu-Goodger C. (2020). Narnaviruses: Novel players in fungal–bacterial symbioses. ISME J..

[49] Hoffman M.T., Arnold A.E. (2010). Diverse bacteria inhabit living hyphae of phylogenetically diverse fungal endophytes. Appl. Environ. Microbiol..

[50] Hoffman M.T., Gunatilaka M.K., Wijeratne K., Gunatilaka L., Arnold A.E. (2013). Endohyphal bacterium enhances production of indole-3-acetic acid by a foliar fungal endophyte. PLoS ONE.

[51] Xie J., Jiang D. (2014). New Insights into mycoviruses and exploration for the biological control of crop fungal diseases. Annu. Rev. Phytopathol..

[52] Zhang H., Xie J., Fu Y., Cheng J., Qu Z., Zhao Z., Cheng S., Chen T., Li B., Wang Q. (2020). A 2-kb mycovirus converts a pathogenic fungus into a beneficial endophyte for *Brassica* protection and yield enhancement. Mol. Plant.

[53] Zhou L., Li X., Kotta-Loizou I., Dong K., Li S., Ni D., Hong N., Wang G., Xu W. (2021). A mycovirus modulates the endophytic and pathogenic traits of a plant associated fungus. ISME J..

[54] Shapira R., Choi G.H., Nuss D.L. (1991). Virus-like genetic organization and expression strategy for a double-stranded RNA genetic element associated with biological control of chestnut blight. EMBO J..

[55] Polashock J.J., Hillman B.I. (1994). A small mitochondrial double-stranded (ds) RNA element associated with a hypovirulent strain of the chestnut blight fungus and ancestrally related to yeast cytoplasmic T and W dsRNAs. Proc. Natl. Acad. Sci. USA.

[56] Yu X., Li B., Fu Y., Jiang D., Ghabrial S.A., Li G., Peng Y., Xie J., Cheng J., Huang J. (2010). A geminivirus-related DNA mycovirus that confers hypovirulence to a plant pathogenic fungus. Proc. Natl. Acad. Sci. USA.

[57] Azhar A., Mu F., Huang H., Cheng J., Fu Y., Hamid M.R., Jiang D., Xie J. (2019). A novel RNA virus related to Sobemoviruses confers hypovirulence on the phytopathogenic fungus *Sclerotinia sclerotiorum*. Viruses.

[58] Kamaruzzaman M., He G., Wu M., Zhang J., Yang L., Chen W., Li G. (2019). A novel partitivirus in the hypovirulent isolate QT5-19 of the plant pathogenic fungus *Botrytis cinerea*. Viruses.

[59] Hao F., Ding T., Wu M., Zhang J., Yang L., Chen W., Li G. (2018). Two novel hypovirulence-associated mycoviruses in the phytopathogenic fungus *Botrytis cinerea*: Molecular characterization and suppression of infection cushion formation. Viruses.

[60] Chu Y.-M., Jeon J.-J., Yea S.-J., Kim Y.-H., Yun S.-H., Lee Y.-W., Kim K.-H. (2020). Double-stranded RNA mycovirus from *Fusarium graminearum*. Appl. Environ. Microbiol..

[61] Márquez L.M., Redman R.S., Rodriguez R.J., Roossinck M.J. (2007). A virus in a fungus in a plant: Three-way symbiosis required for thermal tolerance. Science.

[62] Redman R.S., Sheehan K.B., Stout R.G., Rodriguez R.J., Henson J.M. (2002). Thermotolerance generated by plant/fungal symbiosis. Science.

[63] Shah U.A., Kotta-Loizou I., Fitt B.D.L., Coutts R.H.A. (2020). Mycovirus-induced hypervirulence of *Leptosphaeria biglobosa* enhances systemic acquired resistance to *Leptosphaeria maculans* in *Brassica napus*. Mol. Plant Microbe Interact..

[64] Dewick P.M. (2009). Secondary metabolism: The building blocks and construction mechanisms. Medicinal Natural Products.

[65] Demain A.L., Fang A., Fiechter A. (2000). The natural functions of secondary metabolites. History of Modern Biotechnology I.

[66] Rustamova N., Bozorov K., Efferth T., Yili A. (2020). Novel secondary metabolites from endophytic fungi: Synthesis and biological properties. Phytochem. Rev..

[67] Hacquard S., Kracher B., Hiruma K., Münch P.C., Garrido-Oter R., Thon M.R., Weimann A., Damm U., Dallery J.-F., Hainaut M. (2016). Survival trade-offs in plant roots during colonization by closely related beneficial and pathogenic fungi. Nat. Commun..

[68] Zhou J., Li X., Huang P.-W., Dai C.-C. (2018). Endophytism or saprophytism: Decoding the lifestyle transition of the generalist fungus *Phomopsis liquidambari*. Microbiol. Res..

[69] Zhou J., Li X., Chen Y., Dai C. (2017). *De novo* transcriptome assembly of *Phomopsis liquidambari* provides insights into genes associated with different lifestyles in rice (*Oryza sativa* L.). Front. Plant Sci..

[70] Christianson D.W. (2017). Structural and chemical biology of terpenoid cyclases. Chem. Rev..

[71] Gershenzon J., Dudareva N. (2007). The function of terpene natural products in the natural world. Nat. Chem. Biol..

[72] Matsuda Y., Abe I., Liu H.-W., Begley T.P. (2020). 1.14-Fungal meroterpenoids. Comprehensive Natural Products III.

[73] Farh M.E.-A., Jeon J. (2020). Roles of fungal volatiles from perspective of distinct lifestyles in filamentous fungi. Plant Pathol. J..

[74] Müller A., Faubert P., Hagen M., zu Castell W., Polle A., Schnitzler J.-P., Rosenkranz M. (2013). Volatile profiles of fungi—Chemotyping of species and ecological functions. Fungal Genet. Biol..

[75] Huang M., Sanchez-Moreiras A.M., Abel C., Sohrabi R., Lee S., Gershenzon J., Tholl D. (2012). The major volatile organic compound emitted from Arabidopsis thaliana flowers, the sesquiterpene (E)-β-caryophyllene, is a defense against a bacterial pathogen. New Phytol..

[76] Yamagiwa Y., Inagaki Y., Ichinose Y., Toyoda K., Hyakumachi M., Shiraishi T. (2011). *Talaromyces wortmannii* FS2 emits β-caryphyllene, which promotes plant growth and induces resistance. J. Gen. Plant Pathol..

[77] Bitas V., McCartney N., Li N., Demers J., Kim J.-E., Kim H.-S., Brown K.M., Kang S. (2015). *Fusarium oxysporum* volatiles enhance plant growth via affecting auxin transport and signaling. Front. Microbiol..

[78] Kong H.G., Song G.C., Sim H.-J., Ryu C.-M. (2021). Achieving similar root microbiota composition in neighbouring plants through airborne signalling. ISME J..

[79] Schulz-Bohm K., Gerards S., Hundscheid M., Melenhorst J., de Boer W., Garbeva P. (2018). Calling from distance: Attraction of soil bacteria by plant root volatiles. ISME J..

[80] Schmidt R., Etalo D.W., de Jager V., Gerards S., Zweers H., de Boer W., Garbeva P. (2016). Microbial small talk: Volatiles in fungal–bacterial interactions. Front. Microbiol..

[81] Schmidt R., Jager V.d., Zühlke D., Wolff C., Bernhardt J., Cankar K., Beekwilder J., Ijcken W.v., Sleutels F., Boer W.d. (2017). Fungal volatile compounds induce production of the secondary metabolite sodorifen in *Serratia plymuthica* PRI-2C. Sci. Rep..

[82] Kai M., Piechulla B. (2018). Interspecies interaction of *Serratia plymuthica* 4Rx13 and *Bacillus subtilis* B2g alters the emission of sodorifen. FEMS Microbiol. Lett..

[83] Lin C., Owen S.M., Peñuelas J. (2007). Volatile organic compounds in the roots and rhizosphere of *Pinus* spp.. Soil Biol. Biochem..

[84] de la Porte A., Schmidt R., Yergeau É., Constant P. (2020). A gaseous milieu: Extending the boundaries of the rhizosphere. Trends Microbiol..

[85] Schulz-Bohm K., Geisen S., Wubs E.R.J., Song C., de Boer W., Garbeva P. (2017). The prey’s scent – volatile organic compound mediated interactions between soil bacteria and their protist predators. ISME J..

[86] Ditengou F.A., Müller A., Rosenkranz M., Felten J., Lasok H., van Doorn M.M., Legué V., Palme K., Schnitzler J.-P., Polle A. (2015). Volatile signalling by sesquiterpenes from ectomycorrhizal fungi reprogrammes root architecture. Nat. Commun..

[87] Hung R., Lee S., Bennett J.W. (2013). *Arabidopsis thaliana* as a model system for testing the effect of *Trichoderma* volatile organic compounds. Fungal Ecol..

[88] Lee S., Yap M., Behringer G., Hung R., Bennett J.W. (2016). Volatile organic compounds emitted by *Trichoderma* species mediate plant growth. Fungal Biol. Biotechnol..

[89] Kaddes A., Fauconnier M.-L., Sassi K., Nasraoui B., Jijakli M.-H. (2019). Endophytic fungal volatile compounds as solution for sustainable agriculture. Molecules.

[90] Yang W., Chen X., Li Y., Guo S., Wang Z., Yu X. (2020). Advances in pharmacological activities of terpenoids. Nat. Prod. Commun..

[91] Chassagne F., Cabanac G., Hubert G., David B., Marti G. (2019). The landscape of natural product diversity and their pharmacological relevance from a focus on the Dictionary of Natural Products^®^. Phytochem. Rev..

[92] Huang M., Lu J.-J., Huang M.-Q., Bao J.-L., Chen X.-P., Wang Y.-T. (2012). Terpenoids: Natural products for cancer therapy. Expert. Opin. Investig. Drugs.

[93] Xie S., Wu Y., Qiao Y., Guo Y., Wang J., Hu Z., Zhang Q., Li X., Huang J., Zhou Q. (2018). Protoilludane, illudalane, and botryane sesquiterpenoids from the endophytic fungus *Phomopsis* sp. TJ507A. J. Nat. Prod..

[94] Qu H.-R., Yang W.-W., Zhang X.-Q., Lu Z.-H., Deng Z.-S., Guo Z.-Y., Cao F., Zou K., Proksch P. (2020). Antibacterial bisabolane sesquiterpenoids and isocoumarin derivatives from the endophytic fungus *Phomopsis prunorum*. Phytochem. Lett..

[95] Li S., Chen J.-F., Qin L.-L., Li X.-H., Cao Z.-X., Gu Y.-C., Guo D.-L., Deng Y. (2020). Two new sesquiterpenes produced by the endophytic fungus *Aspergillus fumigatus* from *Ligusticum wallichii*. J. Asian Nat. Prod. Res..

[96] Shi X.-S., Meng L.-H., Li X.-M., Li X., Wang D.-J., Li H.-L., Zhou X.-W., Wang B.-G. (2019). Trichocadinins B–G: Antimicrobial cadinane sesquiterpenes from *Trichoderma virens* QA-8, an endophytic fungus obtained from the medicinal plant *Artemisia argyi*. J. Nat. Prod..

[97] Liu Z., Zhao J.-Y., Sun S.-F., Li Y., Qu J., Liu H.-T., Liu Y.-b. (2019). Sesquiterpenes from an endophytic *Aspergillus flavus*. J. Nat. Prod..

[98] Chen H.-Y., Liu T.-K., Shi Q., Yang X.-L. (2019). Sesquiterpenoids and diterpenes with antimicrobial activity from *Leptosphaeria* sp. XL026, an endophytic fungus in *Panax notoginseng*. Fitoterapia.

[99] Liu S.-S., Jiang J.-X., Huang R., Wang Y.-T., Jiang B.-G., Zheng K.-X., Wu S.-H. (2019). A new antiviral 14-nordrimane sesquiterpenoid from an endophytic fungus *Phoma* sp.. Phytochem. Lett..

[100] Chen S., Li H., Chen Y., Li S., Xu J., Guo H., Liu Z., Zhu S., Liu H., Zhang W. (2019). Three new diterpenes and two new sesquiterpenoids from the endophytic fungus *Trichoderma koningiopsis* A729. Bioorg. Chem..

[101] Wang Y.-N., Xia G.-Y., Wang L.-Y., Ge G.-B., Zhang H.-W., Zhang J.-F., Wu Y.-Z., Lin S. (2018). Purpurolide A, 5/5/5 spirocyclic sesquiterpene lactone in nature from the endophytic fungus *Penicillium purpurogenum*. Org. Lett..

[102] Pang X.-J., Zhang S.-B., Xian P.-J., Wu X., Yang D.-F., Fu H.-Y., Yang X.-L. (2018). Emericellins A and B: Two sesquiterpenoids with an unprecedented tricyclo[4, 4, 2, 1]hendecane scaffold from the liquid cultures of endophytic fungus *Emericella* sp. XL 029. Fitoterapia.

[103] Xu L.-L., Chen H.-L., Hai P., Gao Y., Xie C.-D., Yang X.-L., Abe I. (2019). (+)- and (−)-preuisolactone A: A pair of caged norsesquiterpenoidal enantiomers with a tricyclo[4.4.01,6.02,8]decane carbon skeleton from the endophytic fungus *Preussia isomera*. Org. Lett..

[104] Qi B., Jia F., Luo Y., Ding N., Li S., Shi F., Hai Y., Wang L., Zhu Z.-X., Liu X. (2020). Two new diterpenoids from *Penicillium chrysogenum* MT-12, an endophytic fungus isolated from *Huperzia serrata*. Nat. Prod. Res..

[105] Fan M., Xiang G., Chen J., Gao J., Xue W., Wang Y., Li W., Zhou L., Jiao R., Shen Y. (2020). Libertellenone M, a diterpene derived from an endophytic fungus *Phomopsis* sp. S12, protects against DSS-induced colitis via inhibiting both nuclear translocation of NF-κB and NLRP3 inflammasome activation. Int. Immunopharmacol..

[106] Li W.-Y., Liu Y., Lin Y.-T., Liu Y.-C., Guo K., Li X.-N., Luo S.-H., Li S.-H. (2020). Antibacterial harziane diterpenoids from a fungal symbiont *Trichoderma atroviride* isolated from *Colquhounia coccinea* var. *mollis*. Phytochemistry.

[107] Ariantari N.P., Ancheeva E., Wang C., Mándi A., Knedel T.-O., Kurtán T., Chaidir C., Müller W.E.G., Kassack M.U., Janiak C. (2019). Indole diterpenoids from an endophytic *Penicillium* sp.. J. Nat. Prod..

[108] Zhao J.-C., Wang Y.-L., Zhang T.-Y., Chen Z.-J., Yang T.-M., Wu Y.-Y., Sun C.-P., Ma X.-C., Zhang Y.-X. (2018). Indole diterpenoids from the endophytic fungus *Drechmeria* sp. as natural antimicrobial agents. Phytochemistry.

[109] Ariefta N.R., Kristiana P., Aboshi T., Murayama T., Tawaraya K., Koseki T., Kurisawa N., Kimura K.-i., Shiono Y. (2018). New isocoumarins, naphthoquinones, and a cleistanthane-type diterpene from *Nectria pseudotrichia* 120-1NP. Fitoterapia.

[110] Xu M.-F., Jia O.-Y., Wang S.-J., Zhu Q. (2016). A new bioactive diterpenoid from *Pestalotiopsis adusta*, an endophytic fungus from *Clerodendrum canescens*. Nat. Prod. Res..

[111] Zhao W.-T., Shi X., Xian P.-J., Feng Z., Yang J., Yang X.-L. (2021). A new fusicoccane diterpene and a new polyene from the plant endophytic fungus *Talaromyces pinophilus* and their antimicrobial activities. Nat. Prod. Res..

[112] Long Y., Tang T., Wang L.-Y., He B., Gao K. (2019). Absolute Configuration and biological activities of meroterpenoids from an endophytic fungus of *Lycium barbarum*. J. Nat. Prod..

[113] He Y., Hu Z., Sun W., Li Q., Li X.-N., Zhu H., Huang J., Liu J., Wang J., Xue Y. (2017). Spiroaspertrione A, a bridged spirocyclic meroterpenoid, as a potent potentiator of oxacillin against methicillin-resistant *Staphylococcus aureus* from *Aspergillus* sp. TJ23. J. Org. Chem..

[114] Qiao Y., Zhang X., He Y., Sun W., Feng W., Liu J., Hu Z., Xu Q., Zhu H., Zhang J. (2018). Aspermerodione, a novel fungal metabolite with an unusual 2,6-dioxabicyclo[2.2.1]heptane skeleton, as an inhibitor of penicillin-binding protein 2a. Sci. Rep..

[115] Tang J.-W., Kong L.-M., Zu W.-Y., Hu K., Li X.-N., Yan B.-C., Wang W.-G., Sun H.-D., Li Y., Puno P.-T. (2019). Isopenicins A–C: Two types of antitumor meroterpenoids from the plant endophytic fungus *Penicillium* sp. sh18. Org. Lett..

[116] Chen L., Fan D.-M., Tang J.-W., An T., Li X., Kong L.-M., Puno P.-T., Li Y. (2020). Discovery of isopenicin A, a meroterpenoid as a novel inhibitor of tubulin polymerization. Biochem. Biophys. Res. Commun..

[117] Yang H.-G., Zhao H., Li J.-J., Chen S.-M., Mou L.-M., Zou J., Chen G.-D., Qin S.-Y., Wang C.-X., Hu D. (2017). Phyllomeroterpenoids A-C, multi-biosynthetic pathway derived meroterpenoids from the TCM endophytic fungus *Phyllosticta* sp. and their antimicrobial activities. Sci. Rep..

[118] Deng M., Tan X., Qiao Y., Sun W., Xie S., Shi Z., Lu Y., Chen G., Qi C., Zhang Y. (2021). New secondary metabolites from the endophytic fungus *Aspergillus* sp. from *Tripterygium wilfordii*. Nat. Prod. Res..

[119] do Nascimento J.S., Silva F.M., Magallanes-Noguera C.A., Kurina-Sanz M., dos Santos E.G., Caldas I.S., Luiz J.H.H., Silva E.d.O. (2020). Natural trypanocidal product produced by endophytic fungi through co-culturing. Folia Microbiol..

[120] Qi C., Zhou Q., Gao W., Liu M., Chen C., Li X.-N., Lai Y., Zhou Y., Li D., Hu Z. (2019). Anti-BACE1 and anti-AchE activities of undescribed spiro-dioxolane-containing meroterpenoids from the endophytic fungus *Aspergillus terreus* Thom. Phytochemistry.

[121] Li Q., Chen C., Cheng L., Wei M., Dai C., He Y., Gong J., Zhu R., Li X.-N., Liu J. (2019). Emeridones A–F, a Series of 3,5-demethylorsellinic acid-based meroterpenoids with rearranged skeletons from an endophytic fungus *Emericella* sp. TJ29. J. Org. Chem..

[122] Patil R., Patil S., Maheshwari V., Patil M. (2021). Inhibitory kinetics and mechanism of pentacyclic triterpenoid from endophytic *Colletotrichum gigasporum* against pancreatic lipase. Int. J. Biol. Macromol..

[123] Gupta S., Bhatt P., Chaturvedi P. (2018). Determination and quantification of asiaticoside in endophytic fungus from *Centella asiatica* (L.) Urban. World J. Microbiol. Biotechnol..

[124] Han M., Qin D., Ye T., Yan X., Wang J., Duan X., Dong J. (2019). An endophytic fungus from *Trichoderma harzianum* SWUKD3.1610 that produces nigranoic acid and its analogues. Nat. Prod. Res..

[125] Lutfia A., Munir E., Yurnaliza Y., Basyuni M. (2021). Chemical analysis and anticancer activity of sesterterpenoid from an endophytic fungus *Hypomontagnella monticulosa* Zg15SU and its host *Zingiber griffithii* Baker. Heliyon.

[126] Cheng X., Wei Z., Pu S., Xiang M., Yan A., Zhang Y., Wang X. (2018). Diversity of endophytic fungi of *Paeonia lactiflora* Pallas and screening for fungal paeoniflorin producers. FEMS Microbiol. Lett..

[127] Xin Q., Yuan R., Shi W., Zhu Z., Wang Y., Cong W. (2019). A review for the anti-inflammatory effects of paeoniflorin in inflammatory disorders. Life Sci..

[128] Tazik Z., Rahnama K., White J.F., Soltanloo H., Hasanpour M., Iranshahi M. (2020). LC-MS based identification of stylosin and tschimgine from fungal endophytes associated with *Ferula ovina*. Iran J. Basic Med. Sci..

[129] Valiahdi S.M., Iranshahi M., Sahebkar A. (2013). Cytotoxic activities of phytochemicals from *Ferula* species. Daru.

[130] Liu S., Zhao Y., Heering C., Janiak C., Müller W.E.G., Akoné S.H., Liu Z., Proksch P. (2019). Sesquiterpenoids from the endophytic fungus *Rhinocladiella similis*. J. Nat. Prod..

[131] Wu Y., Chen Y., Huang X., Pan Y., Liu Z., Yan T., Cao W., She Z. (2018). α-Glucosidase inhibitors: Diphenyl ethers and phenolic bisabolane sesquiterpenoids from the mangrove endophytic fungus *Aspergillus flavus* QQSG-3. Mar. Drugs.

[132] Qiu L., Wang P., Liao G., Zeng Y., Cai C., Kong F., Guo Z., Proksch P., Dai H., Mei W. (2018). New eudesmane-type sesquiterpenoids from the mangrove-derived endophytic fungus *Penicillium* sp. J-54. Mar. Drugs.

[133] Chen Y., Zou G., Yang W., Zhao Y., Tan Q., Chen L., Wang J., Ma C., Kang W., She Z. (2021). Metabolites with anti-inflammatory activity from the mangrove endophytic fungus *Diaporthe* sp. QYM12. Mar. Drugs.

[134] Wang P., Yu J.-H., Zhu K., Wang Y., Cheng Z.-Q., Jiang C.-S., Dai J.-G., Wu J., Zhang H. (2018). Phenolic bisabolane sesquiterpenoids from a Thai mangrove endophytic fungus, *Aspergillus* sp. xy02. Fitoterapia.

[135] Deng Q., Li G., Sun M., Yang X., Xu J. (2020). A new antimicrobial sesquiterpene isolated from endophytic fungus *Cytospora* sp. from the Chinese mangrove plant *Ceriops tagal*. Nat. Prod. Res..

[136] Chen S., Ding M., Liu W., Huang X., Liu Z., Lu Y., Liu H., She Z. (2018). Anti-inflammatory meroterpenoids from the mangrove endophytic fungus *Talaromyces amestolkiae* YX1. Phytochemistry.

[137] Long Y., Cui H., Liu X., Xiao Z.e., Wen S., She Z., Huang X. (2017). Acetylcholinesterase inhibitory meroterpenoid from a mangrove endophytic fungus *Aspergillus* sp. 16-5c. Molecules.

[138] Liu Z., Liu H., Chen Y., She Z. (2018). A new anti-inflammatory meroterpenoid from the fungus *Aspergillus terreus* H010. Nat. Prod. Res..

[139] Xu Z., Xiong B., Xu J. (2021). Chemical investigation of secondary metabolites produced by mangrove endophytic fungus *Phyllosticta capitalensis*. Nat. Prod. Res..

[140] Cai R., Jiang H., Mo Y., Guo H., Li C., Long Y., Zang Z., She Z. (2019). Ophiobolin-type sesterterpenoids from the mangrove endophytic fungus *Aspergillus* sp. ZJ-68. J. Nat. Prod..

[141] Song Y.-P., Fang S.-T., Miao F.-P., Yin X.-L., Ji N.-Y. (2018). Diterpenes and sesquiterpenes from the marine algicolous fungus *Trichoderma harzianum* X-5. J. Nat. Prod..

[142] Hsiao G., Chi W.-C., Pang K.-L., Chen J.-J., Kuo Y.-H., Wang Y.-K., Cha H.-J., Chou S.-C., Lee T.-H. (2017). Hirsutane-type sesquiterpenes with inhibitory activity of microglial nitric oxide production from the red alga-derived fungus *Chondrostereum* sp. NTOU4196. J. Nat. Prod..

[143] Song Y.-P., Miao F.-P., Fang S.-T., Yin X.-L., Ji N.-Y. (2018). Halogenated and nonhalogenated metabolites from the marine-alga-endophytic fungus *Trichoderma asperellum* cf44-2. Mar. Drugs.

[144] Neuhaus G.F., Adpressa D.A., Bruhn T., Loesgen S. (2019). Polyketides from marine-derived *Aspergillus porosus*: Challenges and opportunities for determining absolute configuration. J. Nat. Prod..

[145] Xu K., Wei X.-L., Xue L., Zhang Z.-F., Zhang P. (2020). Antimicrobial meroterpenoids and erythritol derivatives isolated from the marine-algal-derived endophytic fungus *Penicillium chrysogenum* XNM-12. Mar. Drugs.

[146] Fang S.-T., Liu X.-H., Yan B.-F., Miao F.-P., Yin X.-L., Li W.-Z., Ji N.-Y. (2021). Terpenoids from the marine-derived fungus *Aspergillus* sp. RR-YLW-12, associated with the red alga *Rhodomela confervoides*. J. Nat. Prod..

[147] Guo Z.-Y., Tan M.-H., Liu C.-X., Lv M.-M., Deng Z.-S., Cao F., Zou K., Proksch P. (2018). Aspergoterpenins A–D: Four new antimicrobial bisabolane sesquiterpenoid derivatives from an endophytic fungus *Aspergillus versicolor*. Molecules.

[148] Tan X., Zhang X., Yu M., Yu Y., Guo Z., Gong T., Niu S., Qin J., Zou Z., Ding G. (2019). Sesquiterpenoids and mycotoxin swainsonine from the locoweed endophytic fungus *Alternaria oxytropis*. Phytochemistry.

[149] Yang H., Liu X.-Y., Zhang P.-L., Gao H.-M., Zhang L.-T., Shen T., Ren D.-M., Lou H.-X., Wang X.-N. (2020). New terpenoids and triketides from culture of the fungus *Botrysphaeria laricina*. Fitoterapia.

[150] Zhang P.-L., Han Y., Zhang L.-T., Wang X.-L., Shen T., Ren D., Lou H., Wang X.-N. (2017). Botrysphones A–C and botrysphins A–F, triketides and diterpenoids from the fungus *Botrysphaeria laricina*. J. Nat. Prod..

[151] Hu H.-T., Liu X.-Y., Zhang P.-L., Gao H.-M., Zhang L.-T., Shen T., Ren D.-M., Lou H.-X., Wang X.-N. (2020). Novel secondary metabolites from the endobryophytic fungus *Botrysphaeria laricina* and their biological activity. Fitoterapia.

[152] Han W.-B., Zhai Y.-J., Gao Y., Zhou H.-Y., Xiao J., Pescitelli G., Gao J.-M. (2019). Cytochalasins and an abietane-type diterpenoid with allelopathic activities from the endophytic fungus *Xylaria* species. J. Agric. Food Chem..

[153] Ma K.-L., Wei W.-J., Li H.-Y., Song Q.-Y., Dong S.-H., Gao K. (2019). Meroterpenoids with diverse ring systems and dioxolanone-type secondary metabolites from *Phyllosticta capitalensis* and their phytotoxic activity. Tetrahedron.

[154] Chen K., Chen C., Guo J., Sun W., Liu J., Yang J., Liu X., Wang J., Luo Z., Zhu H. (2019). Mangiterpenes A–C and 2′,3′-seco-manginoid C, four sesquiterpene/monoterpene–shikimate–conjugated spirocyclic meroterpenoids from *Guignardia mangiferae*. Phytochemistry.

[155] Qin D., Shen W., Wang J., Han M., Chai F., Duan X., Yan X., Guo J., Gao T., Zuo S. (2019). Enhanced production of unusual triterpenoids from *Kadsura angustifolia* fermented by a symbiont endophytic fungus, *Penicillium* sp. SWUKD4.1850. Phytochemistry.

[156] Liu X.-Y., Wang X.-L., Shen T., Ren D.-M., Lou H.-X., Wang X.-N. (2020). Two new triterpenoids from the fungus *Diplodia cupressi*. Nat. Prod. Res..

[157] Zhang W., Lu X., Huo L., Zhang S., Chen Y., Zou Z., Tan H. (2021). Sesquiterpenes and steroids from an endophytic *Eutypella scoparia*. J. Nat. Prod..

[158] Liu M.-T., He Y., Shen L., Hu Z.-X., Zhang Y.-H. (2019). Bipolarins A–H, eight new ophiobolin-type sesterterpenes with antimicrobial activity from fungus *Bipolaris* sp. TJ403-B1. Chin. J. Nat. Med..

[159] Liu M., Sun W., Shen L., Hao X., Al Anbari W.H., Lin S., Li H., Gao W., Wang J., Hu Z. (2019). Bipolaricins A–I, ophiobolin-type tetracyclic sesterterpenes from a phytopathogenic *Bipolaris* sp. fungus. J. Nat. Prod..

[160] Bilal S., Ali L., Khan A.L., Shahzad R., Asaf S., Imran M., Kang S.-M., Kim S.-K., Lee I.-J. (2018). Endophytic fungus *Paecilomyces formosus* LHL10 produces sester-terpenoid YW3548 and cyclic peptide that inhibit urease and α-glucosidase enzyme activities. Arch. Microbiol..

[161] Demain A.L. (1999). Pharmaceutically active secondary metabolites of microorganisms. Appl. Microbiol. Biotechnol..

[162] Bérdy J. (2005). Bioactive microbial metabolites. J. Antibiot..

[163] Gouda S., Das G., Sen S.K., Shin H.-S., Patra J.K. (2016). Endophytes: A treasure house of bioactive compounds of medicinal importance. Front. Microbiol..

[164] Wani M.C., Taylor H.L., Wall M.E., Coggon P., McPhail A.T. (1971). Plant antitumor agents. VI. Isolation and structure of taxol, a novel antileukemic and antitumor agent from *Taxus brevifolia*. J. Am. Chem. Soc..

[165] Stierle A., Strobel G., Stierle D. (1993). Taxol and taxane production by *Taxomyces andreanae*, an endophytic fungus of Pacific yew. Science.

[166] Gangadevi V., Murugan M., Muthumary J. (2008). Taxol determination from *Pestalotiopsis pauciseta*, a fungal endophyte of a medicinal plant. Chin. J. Biotechnol..

[167] Gangadevi V., Muthumary J. (2009). Taxol production by *Pestalotiopsis terminaliae*, an endophytic fungus of *Terminalia arjuna* (arjun tree). Biotechnol. Appl. Biochem..

[168] Kumaran R.S., Choi Y.K., Lee S., Jeon H.J., Jung H., Kim H.J. (2012). Isolation of taxol, an anticancer drug produced by the endophytic fungus, *Phoma betae*. Afr. J. Biotechnol..

[169] Pandi M., Kumaran R.S., Choi Y.K., Kim H.J., Muthumary J. (2011). Isolation and detection of taxol, an anticancer drug produced from *Lasiodiplodia theobromae*, an endophytic fungus of the medicinal plant *Morinda citrifolia*. Afr. J. Biotechnol..

[170] Gokul Raj K., Manikandan R., Arulvasu C., Pandi M. (2015). Anti-proliferative effect of fungal taxol extracted from *Cladosporium oxysporum* against human pathogenic bacteria and human colon cancer cell line HCT 15. Spectrochim Acta A Mol. Biomol. Spectrosc..

[171] Naik B.S. (2019). Developments in taxol production through endophytic fungal biotechnology: A review. Orient. Pharm. Exp. Med..

[172] Torres-Mendoza D., Ortega H.E., Cubilla-Rios L. (2020). Patents on endophytic fungi related to secondary metabolites and biotransformation applications. J. Fungi.

[173] Kumari A., Singh D., Kumar S. (2017). Biotechnological interventions for harnessing podophyllotoxin from plant and fungal species: Current status, challenges, and opportunities for its commercialization. Crit. Rev. Biotechnol..

[174] Montero-Baladía M., Buzón L., Astigarraga I., Delgado P., Iglesias E., Callejo F., López-Veloso M., Minguito J., Fernández-Regueras M., Ubeira M. (2020). Etoposide treatment adjunctive to immunosuppressants for critically ill COVID-19 patients. J. Infect..

[175] Lovetrue B. (2020). The AI-discovered aetiology of COVID-19 and rationale of the irinotecan + etoposide combination therapy for critically ill COVID-19 patients. Med. Hypotheses.

[176] Martino E., Casamassima G., Castiglione S., Cellupica E., Pantalone S., Papagni F., Rui M., Siciliano A.M., Collina S. (2018). *Vinca alkaloids* and analogues as anti-cancer agents: Looking back, peering ahead. Bioorg. Med. Chem. Lett..

[177] Bandara C.J., Siriwardhana A., Karunaratne D.N., Ratnayake Bandara B.M., Wickramasinghe A., Krishnarajah S.A., Karunaratne V. (2021). Production of vincristine and vinblastine by the endophytic fungus *Botryosphaeria laricina* strain (CRS1) is dependent on stimulating factors present in *Catharanthus roseus*. Nat. Prod. J..

[178] El-Sayed E.R. Discovery of the anticancer drug vinblastine from the endophytic *Alternaria alternata* and yield improvement by gamma irradiation mutagenesis. J. Appl. Microbiol..

[179] Palem P.P.C., Kuriakose G.C., Jayabaskaran C. (2015). An endophytic fungus, *Talaromyces radicus*, isolated from *Catharanthus roseus*, produces vincristine and vinblastine, which induce apoptotic cell death. PLoS ONE.

[180] Dhayanithy G., Subban K., Chelliah J. (2019). Diversity and biological activities of endophytic fungi associated with *Catharanthus roseus*. BMC Microbiol..

[181] Kharwar R.N., Verma V.C., Strobel G., Ezra D. (2008). The endophytic fungal complex of *Catharanthus roseus* (L.) G. Don. Curr. Sci..

[182] Pandey S.S., Singh S., Babu C.V., Shanker K., Srivastava N., Shukla A.K., Kalra A. (2016). Fungal endophytes of *Catharanthus roseus* enhance vindoline content by modulating structural and regulatory genes related to terpenoid indole alkaloid biosynthesis. Sci. Rep..

[183] Tang Z., Rao L., Peng G., Zhou M., Shi G., Liang Y. (2011). Effects of endophytic fungus and its elicitors on cell status and alkaloid synthesis in cell suspension cultures of *Catharanthus roseus*. J. Med. Plants Res..

[184] Wall M.E., Wani M.C., Cook C.E., Palmer K.H., McPhail A.T., Sim G.A. (1966). Plant Antitumor agents. I. The isolation and structure of camptothecin, a novel alkaloidal leukemia and tumor inhibitor from *Camptotheca acuminata*. J. Am. Chem. Soc..

[185] Wall M.E., Wani M.C. (1995). Camptothecin and Taxol: Discovery to clinic—Thirteenth Bruce F. Cain Memorial Award Lecture. Cancer Res..

[186] Padmanabha B.V., Chandrashekar M., Ramesha B.T., Gowda H.C.H., Gunaga R.P., Suhas S., Vasudeva R., Ganeshaiah K.N., Shaanker R.U. (2006). Patterns of accumulation of camptothecin, an anti-cancer alkaloid in *Nothapodytes nimmoniana* Graham., in the Western Ghats, India: Implications for identifying high-yielding sources of the alkaloid. Curr. Sci..

[187] Shweta S., Gurumurthy B.R., Ravikanth G., Ramanan U.S., Shivanna M.B. (2013). Endophytic fungi from *Miquelia dentata* Bedd., produce the anti-cancer alkaloid, camptothecine. Phytomedicine.

[188] Aswini A., Soundhari C. (2018). Production of camptothecin from endophytic fungi and characterization by high-performance liquid chromatography and anticancer activity against colon cancer cell line. Asian J. Pharm. Clin. Res..

[189] Shweta S., Zuehlke S., Ramesha B.T., Priti V., Mohana Kumar P., Ravikanth G., Spiteller M., Vasudeva R., Shaanker R.U. (2010). Endophytic fungal strains of *Fusarium solani*, from *Apodytes dimidiata* E. Mey. ex Arn (Icacinaceae) produce camptothecin, 10-hydroxycamptothecin and 9-methoxycamptothecin. Phytochemistry.

[190] Pu X., Qu X., Chen F., Bao J., Zhang G., Luo Y. (2013). Camptothecin-producing endophytic fungus *Trichoderma atroviride* LY357: Isolation, identification, and fermentation conditions optimization for camptothecin production. Appl. Microbiol. Biotechnol..

[191] Kai G., Wu C., Gen L., Zhang L., Cui L., Ni X. (2015). Biosynthesis and biotechnological production of anti-cancer drug camptothecin. Phytochem. Rev..

[192] Godtfredsen W.O., Jahnsen S., Lorck H., Roholt K., Tybring L. (1962). Fusidic acid: A new antibiotic. Nature.

[193] Wilson D.N. (2014). Ribosome-targeting antibiotics and mechanisms of bacterial resistance. Nat. Rev. Microbiol..

[194] Verbist L. (1990). The antimicrobial activity of fusidic acid. J. Antimicrob. Chemother..

[195] Tian C., Gao H., Peng X.-P., Li G., Lou H.-X. (2021). Fusidic acid derivatives from the endophytic fungus *Acremonium pilosum* F47. J. Asian Nat. Prod. Res..

[196] Zhang Z.-B., Du S.-Y., Ji B., Ji C.-J., Xiao Y.-W., Yan R.-M., Zhu D. (2021). New helvolic acid derivatives with antibacterial activities from *Sarocladium oryzae* DX-THL3, an endophytic fungus from Dongxiang wild rice (*Oryza rufipogon* Griff.). Molecules.

[197] Ratnaweera P.B., Williams D.E., de Silva E.D., Wijesundera R.L.C., Dalisay D.S., Andersen R.J. (2014). Helvolic acid, an antibacterial nortriterpenoid from a fungal endophyte, *Xylaria* sp. of orchid *Anoectochilus setaceus* endemic to Sri Lanka. Mycology.

[198] Zhang P., Zhou P.-P., Yu L.-J. (2009). An endophytic taxol-producing fungus from *Taxus* x *media*, *Aspergillus candidus* MD3. FEMS Microbiol. Lett..

[199] Gangadevi V., Muthumary J. (2009). A novel endophytic taxol-producing fungus *Chaetomella raphigera* isolated from a medicinal plant, *Terminalia arjuna*. Appl. Biochem. Biotechnol..

[200] Zhang P., Zhou P.-P., Yu L.-J. (2009). An endophytic taxol-producing fungus from *Taxus media*, *Cladosporium cladosporioides* MD2. Curr. Microbiol..

[201] Yang Y., Zhao H., Barrero R.A., Zhang B., Sun G., Wilson I.W., Xie F., Walker K.D., Parks J.W., Bruce R. (2014). Genome sequencing and analysis of the paclitaxel-producing endophytic fungus *Penicillium aurantiogriseum* NRRL 62431. BMC Genom..

[202] Li J.Y., Sidhu R.S., Ford E.J., Long D.M., Hess W.M., Strobel G.A. (1998). The induction of taxol production in the endophytic fungus—*Periconia* sp. from *Torreya grandifolia*. J. Ind. Microbiol. Biotechnol..

[203] Strobel G., Yang X., Sears J., Kramer R., Sidhu R.S., Hess W.M. (1996). Taxol from *Pestalotiopsis microspora*, an endophytic fungus of *Taxus wallachiana*. Microbiology.

[204] Kumaran R.S., Kim H.J., Hur B.-K. (2010). Taxol promising fungal endophyte, *Pestalotiopsis* species isolated from *Taxus cuspidata*. J. Biosci. Bioeng..

[205] Kumaran R.S., Hur B.-K. (2009). Screening of species of the endophytic fungus *Phomopsis* for the production of the anticancer drug taxol. Biotechnol. Appl. Biochem..

[206] Kumaran R.S., Muthumary J., Hur B.-K. (2008). Taxol from *Phyllosticta citricarpa*, a leaf spot fungus of the angiosperm *Citrus medica*. J. Biosci. Bioengin..

[207] Kumaran R.S., Muthumary J., Hur B.K. (2008). Isolation and identification of taxol, an anticancer drug from *Phyllosticta melochiae* Yates, an endophytic fungus of *Melochia corchorifolia* L.. Food Sci. Biotechnol..

[208] Ran X., Zhang G., Li S., Wang J. (2017). Characterization and antitumor activity of camptothecin from endophytic fungus *Fusarium solani* isolated from *Camptotheca acuminate*. Afr. Health Sci..

[209] Puri S.C., Verma V., Amna T., Qazi G.N., Spiteller M. (2005). An endophytic fungus from *Nothapodytes foetida* that produces camptothecin. J. Nat. Prod..

[210] Kusari S., Zühlke S., Spiteller M. (2009). An endophytic fungus from *Camptotheca acuminata* that produces camptothecin and analogues. J. Nat. Prod..

[211] Rehman S., Shawl A.S., Kour A., Andrabi R., Sudan P., Sultan P., Verma V., Qazi G.N. (2008). An endophytic *Neurospora* sp. from *Nothapodytes foetida* producing camptothecin. Appl. Biochem. Microbiol..

[212] Mohinudeen I.A.H.K., Pandey S., Kanniyappan H., Muthuvijayan V., Srivastava S. (2021). Screening and selection of camptothecin producing endophytes from *Nothapodytes nimmoniana*. Sci. Rep..

[213] Liu K., Ding X., Deng B., Chen W. (2010). 10-Hydroxycamptothecin produced by a new endophytic *Xylaria* sp., M20, from *Camptotheca acuminata*. Biotechnol. Lett..

[214] Musavi S.F., Dhavale A., Balakrishnan R.M. (2015). Optimization and kinetic modeling of cell-associated camptothecin production from an endophytic *Fusarium oxysporum* NFX06. Prep. Biochem. Biotechnol..

[215] Mohinudeen I.A.H.K., Kanumuri R., Soujanya K.N., Shaanker R.U., Rayala S.K., Srivastava S. (2021). Sustainable production of camptothecin from an *Alternaria* sp. isolated from *Nothapodytes nimmoniana*. Sci. Rep..

[216] Kumar A., Patil D., Rajamohanan P.R., Ahmad A. (2013). Isolation, purification and characterization of vinblastine and vincristine from endophytic fungus *Fusarium oxysporum* isolated from *Catharanthus roseus*. PLoS ONE.

[217] Parthasarathy R., Shanmuganathan R., Pugazhendhi A. (2020). Vinblastine production by the endophytic fungus *Curvularia verruculosa* from the leaves of *Catharanthus roseus* and its in vitro cytotoxicity against HeLa cell line. Anal. Biochem..

[218] Eyberger A.L., Dondapati R., Porter J.R. (2006). Endophyte fungal isolates from *Podophyllum peltatum* produce podophyllotoxin. J. Nat. Prod..

[219] Liang Z., Zhang J., Zhang X., Li J., Zhang X., Zhao C. (2015). Endophytic fungus from *Sinopodophyllum emodi* (Wall.) Ying that produces podophyllotoxin. J. Chromatogr. Sci..

[220] Huang J.-X., Zhang J., Zhang X.-R., Zhang K., Zhang X., He X.-R. (2014). *Mucor fragilis* as a novel source of the key pharmaceutical agents podophyllotoxin and kaempferol. Pharm. Biol..

[221] Puri S.C., Nazir A., Chawla R., Arora R., Riyaz-ul-Hasan S., Amna T., Ahmed B., Verma V., Singh S., Sagar R. (2006). The endophytic fungus *Trametes hirsuta* as a novel alternative source of podophyllotoxin and related aryl tetralin lignans. J. Biotechnol..

[222] Yang X., Guo S., Zhang L., Shao H. (2003). Select of producing podophyllotoxin endophytic fungi from podophyllin plant. Nat. Prod. Res. Devel..

[223] Nadeem M., Ram M., Alam P., Ahmad M.M., Mohammad A., Al-Qurainy F., Khan S., Abdin M.Z. (2012). *Fusarium solani*, P1, a new endophytic podophyllotoxin-producing fungus from roots of *Podophyllum hexandrum*. Afr. J. Microbiol. Res..

[224] Kusari S., Spiteller M. (2011). Are we ready for industrial production of bioactive plant secondary metabolites utilizing endophytes?. Nat. Prod. Rep..

[225] Yousefzadi M., Sharifi M., Behmanesh M., Moyano E., Bonfill M., Cusido R.M., Palazon J. (2010). Podophyllotoxin: Current approaches to its biotechnological production and future challenges. Eng. Life Sci..

[226] Gupta S., Chaturvedi P., Kulkarni M.G., Van Staden J. (2020). A critical review on exploiting the pharmaceutical potential of plant endophytic fungi. Biotechnol. Adv..

[227] Meyer V., Andersen M.R., Brakhage A.A., Braus G.H., Caddick M.X., Cairns T.C., de Vries R.P., Haarmann T., Hansen K., Hertz-Fowler C. (2016). Current challenges of research on filamentous fungi in relation to human welfare and a sustainable bio-economy: A white paper. Fungal Biol. Biotechnol..

